# Multicenter Evaluation of the BioFire FilmArray Pneumonia/Pneumonia Plus Panel for Detection and Quantification of Agents of Lower Respiratory Tract Infection

**DOI:** 10.1128/JCM.00128-20

**Published:** 2020-06-24

**Authors:** Caitlin N. Murphy, Randal Fowler, Joan Miquel Balada-Llasat, Amanda Carroll, Hanna Stone, Oluseun Akerele, Blake Buchan, Sam Windham, Amanda Hopp, Shira Ronen, Ryan F. Relich, Rebecca Buckner, Del A. Warren, Romney Humphries, Shelly Campeau, Holly Huse, Suki Chandrasekaran, Amy Leber, Kathy Everhart, Amanda Harrington, Christina Kwong, Andrew Bonwit, Jennifer Dien Bard, Samia Naccache, Cynthia Zimmerman, Barbara Jones, Cory Rindlisbacher, Maggie Buccambuso, Angela Clark, Margarita Rogatcheva, Corrin Graue, Kevin M. Bourzac

**Affiliations:** a University of Nebraska Medical Center, Omaha, Nebraska, USA; b The Ohio State University Wexner Medical Center, Columbus, Ohio, USA; c The Medical College of Wisconsin, Milwaukee, Wisconsin, USA; d Indiana University School of Medicine, Indianapolis, Indiana, USA; e UCLA Health, Los Angeles, California, USA; f Nationwide Children’s Hospital, Columbus, Ohio, USA; g Loyola University Medical Center, Maywood, Illinois, USA; h Children’s Hospital of Los Angeles, Los Angeles, California, USA; i MRIGlobal, Palm Bay, Florida, USA; j BioFire Diagnostics LLC, Salt Lake City, Utah, USA; UNC School of Medicine

**Keywords:** respiratory pathogens, respiratory viruses, syndromic panel

## Abstract

The ability to provide timely identification of the causative agents of lower respiratory tract infections can promote better patient outcomes and support antimicrobial stewardship efforts. Current diagnostic testing options include culture, molecular testing, and antigen detection. These methods may require collection of various specimens, involve extensive sample treatment, and can suffer from low sensitivity and long turnaround times. This study assessed the performance of the BioFire FilmArray Pneumonia Panel (PN panel) and Pneumonia Plus Panel (PNplus panel), an FDA-cleared sample-to-answer assay that enables the detection of viruses, atypical bacteria, bacteria, and antimicrobial resistance marker genes from lower respiratory tract specimens (sputum and bronchoalveolar lavage [BAL] fluid). Semiquantitative results are also provided for the bacterial targets. This paper describes selected analytical and clinical studies that were conducted to evaluate performance of the panel for regulatory clearance. Prospectively collected respiratory specimens (846 BAL and 836 sputum specimens) evaluated with the PN panel were also tested by quantitative reference culture and molecular methods for comparison. The PN panel showed a sensitivity of 100% for 15/22 etiologic targets using BAL specimens and for 10/24 using sputum specimens. All other targets had sensitivities of ≥75% or were unable to be calculated due to low prevalence in the study population. Specificity for all targets was ≥87.2%, with many false-positive results compared to culture that were confirmed by alternative molecular methods. Appropriate adoption of this test could provide actionable diagnostic information that is anticipated to impact patient care and antimicrobial stewardship decisions.

## Abstract (precis)

The ability to provide timely identification of the causative agents of lower respiratory tract infections can promote better patient outcomes and support antimicrobial stewardship efforts. Current diagnostic testing options include culture, molecular testing, and antigen detection. These methods may require collection of various specimens, involve extensive sample treatment, and can suffer from low sensitivity and long turnaround times. This study assessed the performance of the BioFire FilmArray Pneumonia Panel (PN panel) and Pneumonia Plus Panel (PNplus panel), an FDA-cleared sample-to-answer assay that enables the detection of viruses, atypical bacteria, bacteria, and antimicrobial resistance marker genes from lower respiratory tract specimens (sputum and bronchoalveolar lavage [BAL] fluid).

## INTRODUCTION

Lower respiratory tract infections (LRTI) are clinical conditions that arise throughout the population. Community-acquired pneumonia is estimated to be the most common cause of infectious disease-related mortality in the United States and globally (1, 2) and is a leading cause of hospital and emergency room visits. The highest morbidity and mortality of these illnesses are frequently seen in the elderly, children <5 years of age, and the immunocompromised (3). Pneumonia-like illness is also a frequent hospital-acquired infection that can result in increased mortality and unnecessary economic burden (4).

Bacteria and viruses are the most common etiologies of lower respiratory tract infections. Patients with viral pneumonia may be managed differently than those with bacterial infections, but due to similarities in clinical presentation and symptomatology, it is not possible to distinguish viral from bacterial infections without the aid of laboratory diagnostic testing. Rapid diagnostics for specific entities (Streptococcus pneumoniae and respiratory syncytial virus [RSV]) and host markers (procalcitonin) exist for the detection of common viral and bacterial illness and/or to aid in distinguishing bacterial from viral infections (5–7). These methods alone are frequently inadequate as a means to diagnose and treat pneumonia (8, 9).

Rapid resolution of the etiology of lower respiratory tract infections can aid in the ability to ensure that appropriate antimicrobial therapy is initiated and that patients are put on the appropriate infection control precautions and to prevent unnecessary downstream testing. In adult populations, broad-spectrum antibiotics are often initiated before bacterial culture results are available, if there is suspicion of bacterial pneumonia or if the patient requires ICU admission (6). In children, viral entities are the most frequent cause of pneumonia, and directed antiviral therapy is recommended for severely ill patients (10). Rapid detection of the causative agent of respiratory infection, coupled with detection of prominent markers of antibiotic resistance, can aid in limiting unnecessary broad-spectrum antimicrobial treatment.

The BioFire FilmArray Pneumonia Panel (PN panel) and Pneumonia Plus Panel (PNplus panel) (BioFire Diagnostics, LLC, Salt Lake City, UT) were designed to provide a means of rapidly detecting nucleic acids from common agents of community- and hospital-acquired lower respiratory tract infections (Table 1). The panel integrates nucleic acid extraction, reverse transcription, and nested multiplex PCR amplification for 8 (PN panel) or 9 (PNplus panel) viruses, 18 bacteria (including 3 atypical bacteria associated with community-acquired pneumonia), and 7 antimicrobial resistance (AMR) genes. The PN panel and PNplus panel test reagents are identical, with results for Middle East respiratory syndrome coronavirus (MERS-CoV) masked by the software for the PN panel version; for simplicity, the tests are referred to collectively as the PN panel throughout this paper except where a distinction is required. The device is intended for use with sputum-like specimens (expectorated or induced sputum and endotracheal aspirates [ETA]) and bronchoalveolar lavage (BAL) specimens tested directly, without pretreatment. In addition to nucleic acid detection, the panel is able to provide a semiquantitative estimate of abundance for 15 of the bacterial targets (reported in log_10_ increments from 10^4^ to 10^7^ genomic copies/ml). All testing is done in the closed sample-to-answer FilmArray system, which provides automated analysis and results in about 75 min.

**TABLE 1:** Targets identified by the BioFire FilmArray PN panel

Type	Target
Viruses	Adenovirus
	Coronavirus
	Human metapneumovirus
	Human rhinovirus/enterovirus
	Influenza A virus
	Influenza B virus
	Parainfluenza virus
	Respiratory syncytial virus
	Middle East respiratory syndrome coronavirus^*a*^
Bacteria^*e*^	Acinetobacter calcoaceticus-A. baumannii complex
	Enterobacter cloacae complex
	Escherichia coli
	Haemophilus influenzae
	Klebsiella aerogenes
	Klebsiella oxytoca
	Klebsiella pneumoniae group
	Moraxella catarrhalis
	*Proteus* spp.
	Pseudomonas aeruginosa
	Serratia marscens
	Staphylococcus aureus
	Streptococcus agalactiae
	Streptococcus pneumoniae
	Streptococcus pyogenes
Atypical bacteria	Chlamydia pneumoniae
	Legionella pneumophila
	Mycoplasma pneumoniae
Antimicrobial resistance genes	*mecA*/*mecC* and MREJ^*b*^
	KPC^*c*^
	NDM^*c*^
	OXA-48-like^*d*^
	VIM^*c*^
	IMP^*c*^
	CTX-M^*c*^

a MERS-CoV results are reported only in the BioFire PNplus panel product.

b Reported when S. aureus is also detected.

c Reported when A. calcoaceticus-A. baumannii complex, E. cloacae complex, E. coli, K. aerogenes, K. oxytoca, K. pneumoniae group, Proteus spp., P. aeruginosa, or S. marcescens is also detected.

d Reported when E. cloacae complex, E. coli, K. aerogenes, K. oxytoca, K. pneumoniae group, *Proteus* spp., or S. marcescens is also detected.

e Semiquantitative results from 10^4^ to ≥10^7^ are provided for these analytes.

Here, we report on studies performed to characterize the linearity and accuracy of the semiquantitative results provided for the bacteria detected by the PN panel as well as a multicenter prospective study, where the performance of the panel was evaluated in comparison to several reference methods that included conventional and quantitative culture and molecular detection.

## MATERIALS AND METHODS

### Contrived samples for linearity and accuracy validation.

Dilutions of contrived BAL samples containing cultured bacterial isolates in a matrix of sterile physiological saline and 20 ng/μl human genomic DNA were tested repeatedly with the PN panel (90 replicates) to assess both the linearity and accuracy of the test’s semiquantitative bin results. Each bacterium was tested at six concentrations in 1-log intervals extending above and below the reportable range of the panel (<10^3.5^ through ≥10^7^ copies/ml). The reference or input concentration of bacterial genomic DNA (copies per milliliter) in each contrived sample was determined by digital PCR (dPCR). The nucleic acid quantification method implemented mechanical and chemical lysis of each cultured bacterium (bead-beating on the Disruptor Genie [Scientific Industries] at approximately 3,000 rpm for 3 min and Magna Pure bacterial lysis/binding buffer) followed by total nucleic acid extraction and purification using the Roche Magna Pure LC 2.0 platform (Roche Diagnostics, Indianapolis, IN). Extracts were quantified by dPCR (QuantStudio 3D digital PCR system; Thermo Fisher Scientific, Waltham, MA) using single-copy target assays with sequence-specific fluorescent probes and QuantStudio 3D dPCR Master Mix, according to the QuantStudio user guide. The Thermo Fisher QuantStudio 3D Analysis suite was used to calculate the concentration (copies per milliliter) of the DNA in the culture based upon negative well fractions and the partition volume.

### Contrived polymicrobial clinical specimens.

Individual prescreened and PN panel analyte-negative BAL and sputum specimens were multispiked with Acinetobacter baumannii, Enterobacter cloacae, and Escherichia coli or with Klebsiella oxytoca, Proteus mirabilis, and Serratia marcescens at various concentrations (10^4^ copies/ml, 10^5.5^ copies/ml, or 10^7^ copies/ml) (Table 2). Six different organism-concentration combinations were prepared in replicates of 10 for both sample types (120 contrived samples in total).

**TABLE 2:** Contrived polymicrobial clinical specimens

Sample set	Organism	Spike level (copies/ml)^*a*^	Sample	No. with BioFire PN panel result		
				10^4^ copies/ml	10^6^ copies/ml	≥10^7^ copies/ml
1	A. baumannii	10^4^	BAL	10	0	0
			Sputum	10	0	0
	E. cloacae	10^5.5^	BAL	0	6	4
			Sputum	0	10	0
	E. coli	10^7^	BAL	0	0	10
			Sputum	0	0	10
2	E. cloacae	10^4^	BAL	10	0	0
			Sputum	10	0	0
	E. coli	10^5.5^	BAL	0	10	0
			Sputum	0	10	0
	A. baumannii	10^7^	BAL	0	0	10
			Sputum	0	0	10
3	E. coli	10^4^	BAL	10	0	0
			Sputum	10	0	0
	A. baumannii	10^5.5^	BAL	0	10	0
			Sputum	0	10	0
	E. cloacae	10^7^	BAL	0	0	10
			Sputum	0	0	10
4	K. oxytoca	10^4^	BAL	10	0	0
			Sputum	10	0	0
	P. mirabilis^*b*^	10^5.5^	BAL	0	10	0
			Sputum	0	10	0
	S. marcescens	10^7^	BAL	0	0	10
			Sputum	0	0	10
5	P. mirabilis	10^4^	BAL	10	0	0
			Sputum	10	0	0
	S. marcescens	10^5.5^	BAL	0	10	0
			Sputum	0	10	0
	K. oxytoca	10^7^	BAL	0	0	10
			Sputum	0	0	10
6	S. marcescens	10^4^	BAL	10	0	0
			Sputum	10	0	0
	K. oxytoca	10^5.5^	BAL	0	10	0
			Sputum	0	10	0
	P. mirabilis	10^7^	BAL	0	0	9^*a*^
			Sputum	0	0	10

a Levels of 10^4^, 10^5.5^, and 10^7^ are considered low, medium, and high, respectively.

b P. mirabilis in one BAL specimen was reported as “not detected” by the BioFire PN panel.

### Clinical specimens.

Sputum specimens (including ETA) and BAL specimens (including mini-BAL specimens, which do not require bronchoscopy) were enrolled at eight geographically distinct U.S. sites (Medical College of Wisconsin, Milwaukee, WI; Children’s Hospital of Los Angeles, Los Angeles, CA; The Ohio State University Wexner Medical Center, Columbus, OH; Nationwide Children’s Hospital, Columbus, OH; Loyola University Medical Center, Maywood, IL; Indiana University School of Medicine, Indianapolis, IN; University of Nebraska Medical Center, Omaha, NE; and University of California Los Angeles Health, Los Angeles, CA) from October 2016 until July 2017. Residual specimens from subjects of all ages that had been submitted to the laboratory for bacterial culture were enrolled if they met the following criteria: sufficient volume (at least 1.5 ml), no processing or pretreatment (i.e., “native” specimens), and the ability to be enrolled within 24 h of collection. Sites followed their own procedures and criteria for determining whether specimens were of appropriate quality for culture workup; specimens that were rejected (and thus did not have an associated standard-of-care [SOC] culture result) were not eligible for enrollment in the study. A waiver of the requirement for informed consent was obtained from the institutional review board (IRB) at each study site for the use of residual specimens and in order to collect subject information from the medical records. Clinical and demographic data were collected, including hospitalization status at the time of specimen collection, the results of the clinician-ordered SOC culture, subject sex, and subject age category. Sites were instructed to enroll specimens in the morning as the first study activity of the day so that all specimen aliquoting, shipping, freezing, and PN panel testing were performed in temporal proximity. Specimens were coded or pseudonymized by the study enroller, thoroughly mixed by vortexing, and then pipetted into various aliquots for testing. One aliquot was used for testing on-site with the PN panel. An additional aliquot was shipped overnight at refrigeration temperature to a central reference laboratory (MRIGlobal, Palm Bay, FL) for reference culture, and finally, several aliquots were immediately frozen for molecular comparator testing.

### PN panel testing.

This study was conducted with an investigational-use-only (IUO) version of the PN panel that is identical to the commercial (i.e., FDA-cleared, CE-marked) *in vitro* diagnostic (IVD) version. All specimen handling occurred in a biosafety cabinet with operators wearing appropriate personal protective equipment, preparing one specimen at a time, and cleaning between specimens, all according to the manufacturer’s instructions (11). In contrast to other BioFire FilmArray test panels, which use a transfer pipette for specimen loading, specimens are introduced into the PN panel test with a provided flocked swab. This facilitates recovery of organisms from viscous lower respiratory tract specimens. Briefly, the native specimen was collected on the provided sample transfer swab (approximately 200 μl) and placed in sample buffer within the FilmArray injection vial (FAIV). The sample swab was broken off inside the FAIV at a prescored breakpoint. After the lid was closed, the FAIV was gently inverted three times to facilitate organism release, and then the contents were injected into the PN panel pouch before testing with the FilmArray instrument. The PN panel test consists of automated nucleic acid extraction, reverse transcription, nucleic acid amplification, and automated results analysis in approximately 75 min per run (i.e., per specimen). If either of two internal controls fails, the software automatically provides a result of “invalid” for all panel analytes. Viruses and atypical bacteria are reported qualitatively as “detected” or “not detected” (an “equivocal” result is also possible for MERS-CoV on the PNplus panel). The AMR genes are also reported qualitatively (“detected” or “not detected”), but only if one or more applicable bacteria (i.e., potential carriers of the AMR gene) are also detected in the sample (Table 1, footnotes c and d); if no applicable bacteria are detected, the AMR gene results are reported as “N/A” (not applicable). For 15 bacterial targets, the BioFire PN panel calculates an approximate quantity of the gene target (i.e., bacterial DNA, in copies per milliliter) based on real-time amplification curves for the bacterial assays relative to a quantified internal reference standard manufactured into each PN panel test cartridge. The assays are designed to amplify genes that are present in single copies within the chromosome of the target bacterium and thus to estimate a concentration of targeted bacterial genome equivalents in the specimen. The calculated value is rounded to the nearest 10*^n^* value and reported as a bin result (10^4^, 10^5^, 10^6^, or ≥10^7^ genomic copies/ml). Assays with no measurable amplification or a calculated value below 10^3.5^ (3,162) copies/ml are considered negative and reported as “not detected.”

### Comparator testing.

**(i) Standard-of-care culture.** All eight study sites followed their own standard procedures to determine SOC culture results, independent of the study. While the methods for culture of lower respiratory tract specimens are relatively standardized, each site (and sometimes technicians within a site) had variation with respect to whether and how organisms were reported (12). Results were obtained from chart review of subject medical information.

**(ii) Quantitative reference culture.** A central reference laboratory (MRIGlobal, Palm Bay, FL) was used to perform quantitative reference culture (qRefCx). This approach was similar to the method that the study sites use for routine standard-of-care (SOC) culture; however, different sites’ SOC protocols varied. The reference lab was used in order to standardize the plating protocol and results and, in particular, to ensure that quantitative results were obtained over the reportable range of the PN panel for both specimen types. Aliquots of enrolled specimens were shipped overnight at refrigeration temperature (on ice) to the central reference laboratory. Specimens were excluded if they did not arrive at the reference lab with sufficient time to be processed for culture within one calendar day of enrollment or if they were no longer at refrigeration temperature upon arrival. BAL and sputum specimens were treated the same except that sputum specimens were pretreated with an equal volume of SnotBuster (Copan, Murrieta, CA) mucolytic reagent to reduce viscosity before plating.

Specimens were streaked onto four different media (blood agar, chocolate agar, MacConkey agar, and Columbia colistin-nalidixic acid [CNA] agar) at four different concentrations: 10 μl and 1 μl of both undiluted and 1:100-diluted specimen. Plates were incubated at 35°C and inspected for growth at 24 and 48 h. Quantity was determined by counting colonies of each unique morphology on the plate type with the most robust growth of that morphology and at the dilution with 20 to 200 colonies of that morphology. If an organism was observed on multiple plates, the highest quantification value was used. Identification was described first by colony morphology and then confirmed by Vitek 2 ID (bioMérieux, Durham, NC) following isolation and subculturing. Vitek 2 was also used for phenotypic antimicrobial susceptibility testing (AST). Glycerol stocks of relevant bacterial isolates were prepared for molecular AMR gene testing and discrepancy investigation.

**(iii) Real-time PCR and sequencing.** Total nucleic acids were extracted from clinical specimens using a Magna Pure LC 2.0 instrument. Atypical bacteria and viruses were tested with two well-validated nested PCR assays; AMR genes were tested with a single assay. Whenever possible, the comparator PCR assays targeted different genes (or different regions of the same gene) than are targeted by the PN panel assays. Assays were designed to generate amplicons that would provide sufficient sequence information for conclusive analyte identification (between 100 and 200 bp). A sequence-confirmed positive result from either assay was considered positive for a given analyte. Validation testing demonstrated that most assays (at least one or both per analyte in both BAL and sputum sample types) had a limit of detection (LoD) that was within at least 5-fold that of the PN panel, which was considered “equivalent” sensitivity. All specimens were assumed to be negative for MERS-CoV, as it was not circulating in the United States during the time of enrollment for the study; no comparator testing was performed for this analyte.

**Results and discrepant analysis.** A PN panel result was considered a true positive (TP) or true negative (TN) when it agreed with the result from the comparator method. Discrepant analysis was performed when results were discordant, i.e., false-positive (FP) or false-negative (FN) results. When sufficient specimen volume was available, discordant specimens were investigated using a combination of retesting with the PN panel or comparator methods as well as testing with additional, independent molecular assays. Note that the performance data for positive percent agreement (PPA) and negative percent agreement (NPA) presented in this paper consist of unresolved data as presented in the package insert for the commercial test; discrepancy investigation is provided but was not used to recalculate performance data.

### Statistical analysis.

The exact binomial two-sided 95% confidence intervals (95% CI) were calculated for performance measures according to the Wilson score method (13).

## RESULTS

### Linearity and accuracy of PN panel semiquantitative bin results for bacteria.

**FIG 1:**
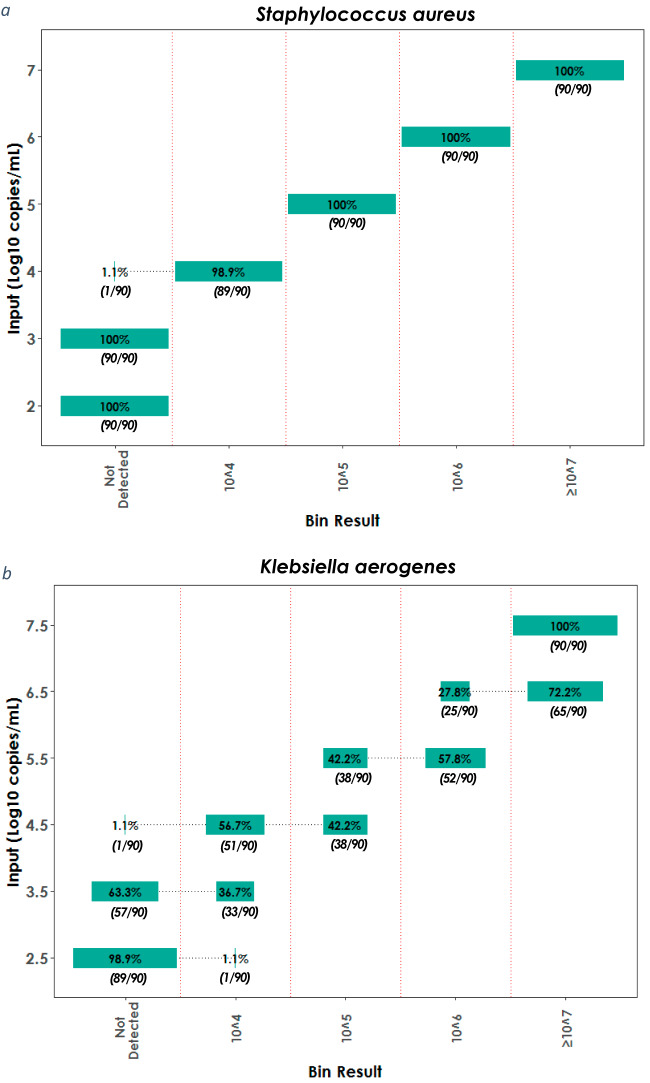
BioFire PN panel bin result linearity and accuracy. Contrived BAL samples containing S. aureus (a) or K. aerogenes (b) were quantified by dPCR and spiked at 1-log serial dilutions spanning the range of BioFire PN panel bin reporting. The reported percent (of 90 replicates) is graphed as a bar in each bin at each dilution tested.

Each PN panel bacterial assay was designed to be efficient and linear and to provide accurate bin results within ±0.5 log_10_ copies/ml of the input concentration over a reportable range of 10^4^ to >10^7^ copies/ml. The linearity and accuracy of the assays were validated by testing a 1-log dilution series of contrived samples containing each bacterium detected by the panel. Results for Staphylococcus aureus and Klebsiella aerogenes are shown in Fig. 1 as representative of Gram-positive and Gram-negative organisms, respectively; additional data for all other bacteria can be found in the product instructions for use (11). Each of the samples at six concentrations was tested repeatedly (90 pouches) and the bin results of the test (in copies per milliliter) were compared to the input concentration (also in copies per milliliter) of the sample.

Contrived samples containing Staphylococcus aureus at input concentrations of 10^2^, 10^3^, 10^4^, 10^5^, 10^6^, and 10^7^ copies/ml (representing the “middle” of the bin) were tested with the PN panel, and S. aureus was detected in 99% (359/360) of all replicates with concentrations within the reportable range of 10^4^ copies/ml and higher (Fig. 1a). Over the dilution series, the semiquantitative bin result changed linearly, in direct proportion to the change in sample input concentration (e.g., an increase in concentration of 1 log_10_ copies/ml generated a change in bin result equivalent to 1 log_10_ copies/ml). In addition, the semiquantitative bin result reflected the sample input concentration within the stated ±0.5-log_10_-copies/ml accuracy of the panel. For example, the sample input concentration of 10^5^ copies/ml has an expected accuracy range of 10^4.5^ to 10^5.5^, and an accurate bin result of 10^5^ copies/ml was reported by the PN panel for 100% of the sample replicates tested.

Contrived samples containing K. aerogenes were tested at input concentrations of 10^2.5^, 10^3.5^, 10^4.5^, 10^5.5^, 10^6.5^, and 10^7.5^ copies/ml (representing the “edge” of the bin), and K. aerogenes was detected in 87% (392/450) of all replicates at a concentration of 10^3.5^ copies/ml and higher (Fig. 1b). The semiquantitative bin result changed linearly, in direct proportion to the change in sample concentration, though at each concentration except the highest two bins, results were reported in variable proportions over the 90 replicates. Although more than one bin result was reported in different replicates of the same input concentration, each bin result was accurate relative to the input concentration within ±0.5 log_10_ copies/ml. For example, the sample input concentration of 10^5.5^ copies/ml has an accuracy range of 10^5.0^ to 10^6.0^ copies/ml (spanning two bins). The PN panel provided an accurate bin result of 10^5^ copies/ml in 42.2% of the replicates tested at this concentration with an equally accurate bin result of 10^6^ copies/ml for the remaining 57.8% of the sample replicates tested.

### Semiquantification in contrived polymicrobial specimens.

The organisms are reported at a semiquantitative level, and thus, the accuracy of the expected relative rank order among contrived polymicrobial specimens (low, medium, and high) was tested. In 60 (100%) contrived sputum specimens and in 55 of 60 (91.6%) BAL specimens (Table 2), the correct relative rank was observed. Four specimens in BAL sample set 1 that were spiked with E. cloacae at a medium level of 10^5.5^ genomic copies/ml were reported by the PN panel as “detected” at ≥10^7^ (high) instead of the expected level of 10^6^. All other organisms in these four samples were reported at the correct level, and this organism was reported at the correct level in all 10 specimens of the corresponding sputum sample set, set 1. One additional specimen in BAL sample set 6 that was spiked with P. mirabilis at 10^7^ genomic copies/ml was unexpectedly negative, but the other two organisms in the specimen were reported correctly. All other results for P. mirabilis in all other samples were reported correctly.

### Clinical demographics.

A total of 904 BAL specimens (821 BAL and 83 mini-BAL specimens) and 925 sputum specimens (478 sputum specimens and 447 ETA) were collected for the prospective clinical study from eight U.S. clinical sites. Fifty-eight BAL and 89 sputum specimens were excluded after enrollment. The most common reasons for specimen exclusion was that reference culture could not be performed within the required time frame (as described in Materials and Methods). Sex, age, and patient care setting (hospitalized, outpatient, or emergency department [ED]) were recorded for all subjects from whom specimens were enrolled. The clinical demographics associated with the 1,682 valid enrolled specimens are presented in Table 3. There were slightly more specimens collected from male subjects (480 BAL [57%] and 481 sputum [58%] specimens) than from female subjects (366 BAL [43%] and 355 sputum [42%] specimens). The age distribution of subjects with enrolled specimens included pediatric patients less than 18 years of age (50 BAL [6%] and 245 sputum [29%] specimens), adults between 18 and 65 years of age (540 BAL [64%] and 370 sputum [44%] specimens), and adults older than 65 years of age (255 BAL [30%] and 221 sputum [26%] specimens). The subject’s age could not be determined for one BAL specimen enrolled. The majority of specimens (80%) were collected from hospitalized subjects (666/846 [79%] BAL and 682/836 [82%] sputum specimens), with outpatient and ED collections accounting for 19% of BAL specimens (159/845 outpatient [19%] and 21/845 ED [2.5%] specimens) and 18% of sputum specimens (73/836 outpatient [8.5%] and 81/836 ED [10%] specimens).

**TABLE 3:** Study demographics (valid specimens only)

Specimen and patient type	No. of subjects by age (yr):					Total
	<5	6 to 17	18–34	35 to 65	>65	
BAL						
Inpatient	8	18	61	366	212	666
Outpatient	15	8	5	93	38	159
Emergency department	0	1	4	11	5	21
Total	23	27	70	470	255	846
Sputum						
Inpatient	102	64	68	252	196	682
Outpatient	13	21	7	18	14	73
Emergency department	23	22	11	14	11	81
Total	138	107	86	284	221	836

### Test performance and summary of the PN panel.

In the prospective clinical evaluation, a total of 1,796 of 1,798 PN panel test runs (889 BAL and 909 sputum specimens) were completed on the first attempt, for an overall instrument success rate of 99.9%. Of the 1,796 completed runs, 1,764 (98.2%) produced a valid result (i.e., successful pouch controls). Twenty-eight of the 32 specimens with control failures had sufficient volume for retesting and were able to be retested within study-defined time interval (without specimen dilution or manipulation); 25 produced a valid result on the single retest. The pouch controls failed a second time for the remaining three specimens, and there was no further specimen volume for testing.

Of the valid runs, the PN panel detected at least one analyte in 413 of 846 BAL specimens and in 602 of 836 sputum specimens for an overall positivity rate of 48.8 and 72.0%, respectively (Table 4). Codetections were observed in 37.8% (156/413) of BAL specimens and 56.5% (340/836) of sputum specimens. The most commonly detected analytes were Staphylococcus aureus, Pseudomonas aeruginosa, Haemophilus influenzae, and human rhinovirus/enterovirus (HRV/EV), which were found in 320 (19%), 234 (13.9%), 189 (11.2%), and 176 (10.5%) specimens, respectively. All other analytes were detected in fewer than 105 (6.2%) specimens. The overall prevalence of each analyte stratified by collection location is shown in Table 5.

**TABLE 4:** Multiple analyte detections by the BioFire PN panel

BioFire PN panel result	BAL (*n* = 846)		Sputum (*n* = 836)	
	No. detected	% of total (% of positives)	No. detected	% of total (% of positives)
Total positive specimens	413	48.8 (100)	602	72.0 (100)
One analyte result	257	30.4 (62.2)	262	31.3 (43.5)
Two analyte results	105	12.4 (25.4)	178	21.3 (29.6)
Three analyte results	28	3.3 (6.8)	85	10.2 (14.1)
Four analyte results	20	2.4 (4.8)	42	5.0 (7.0)
Five analyte results	2	0.2 (0.5)	23	2.8 (3.8)
Six or more analyte results	1	0.1 (0.2)	12	1.4 (2.0)

**TABLE 5:** Total number of BioFire PN panel detections for each analyte by specimen collection location and specimen type

Target	No. (%) of detections in:							
	BAL (*n* = 846)				Sputum (*n* = 836)			
	Hospitalized (*n* = 666)	Outpatient (*n* = 159)	ED (*n* = 21)	Total (*n* = 846)	Hospitalized (*n* = 682)	Outpatient (*n* = 73)	ED (*n* = 81)	Total (*n* = 836)
Bacteria								
A. calcoaceticus-A. baumannii complex	6 (0.9)	0 (0)	1 (4.8)	7 (0.8)	17 (2.5)	6 (8.2)	5 (6.2)	28 (3.3)
E. cloacae complex	22 (3.3)	0 (0)	1 (4.8)	23 (2.7)	25 (3.7)	5 (6.8)	2 (2.5)	32 (3.8)
E. coli	18 (2.7)	0 (0)	2 (9.5)	20 (2.4)	38 (5.6)	2 (2.7)	8 (9.9)	48 (5.7)
H. influenzae	61 (9.2)	17 (10.7)	4 (19.0)	82 (9.7)	84 (12.3)	13 (17.8)	10 (12.3)	107 (12.8)
K. aerogenes	12 (1.8)	1 (0.6)	0 (0)	13 (1.5)	10 (1.5)	0 (0)	2 (2.5)	12 (1.4)
K. oxytoca	10 (1.5)	1 (0.6)	0 (0)	11 (1.3)	14 (2.1)	3 (4.1)	2 (2.5)	19 (2.3)
K. pneumoniae group	23 (3.5)	3 (1.9)	1 (4.8)	27 (3.2)	54 (7.9)	6 (8.2)	5 (6.2)	65 (7.8)
M. catarrhalis	18 (2.7)	11 (6.9)	0 (0)	29 (3.4)	45 (6.6)	16 (21.9)	14 (17.3)	75 (9.0)
*Proteus* spp.	9 (1.4)	0 (0)	0 (0)	9 (1.1)	10 (1.5)	5 (6.8)	8 (9.9)	23 (2.8)
P. aeruginosa	58 (8.7)	13 (8.2)	3 (14.3)	74 (8.7)	106 (15.5)	28 (38.4)	26 (32.1)	160 (19.1)
S. marcescens	9 (1.4)	2 (1.3)	1 (4.8)	12 (1.4)	27 (4.0)	10 (13.7)	16 (19.8)	53 (6.3)
S. aureus	100 (15)	10 (6.3)	6 (28.6)	116 (13.7)	152 (22.3)	26 (35.6)	26 (32.1)	204 (24.4)
S. agalactiae	21 (3.2)	3 (1.9)	1 (4.8)	25 (3.0)	28 (4.1)	7 (9.6)	8 (9.9)	43 (5.1)
S. pneumoniae	21 (3.2)	8 (5.0)	0 (0)	29 (3.4)	35 (5.1)	4 (5.5)	12 (14.8)	51 (6.1)
S. pyogenes	5 (0.8)	2 (1.3)	1 (4.8)	8 (0.9)	8 (1.2)	1 (1.4)	2 (2.5)	11 (1.3)
Antimicrobial resistance genes								
*mecA*/*mecC* and MREJ	43 (6.5)	1 (0.6)	2 (9.5)	46 (5.4)	81 (11.9)	14 (19.2)	12 (14.8)	107 (12.8)
KPC	2 (0.3)	0 (0)	1 (4.8)	3 (0.4)	6 (0.9)	1 (1.4)	0 (0)	7 (0.8)
NDM	1 (0.2)	0 (0)	0 (0)	1 (0.1)	0 (0)	0 (0)	0 (0)	0 (0)
OXA-48-like	0 (0)	0 (0)	0 (0)	0 (0)	0 (0)	0 (0)	0 (0)	0 (0)
VIM	0 (0)	0 (0)	0 (0)	0 (0)	2 (0.3)	0 (0)	0 (0)	2 (0.2)
IMP	0 (0)	0 (0)	0 (0)	0 (0)	0 (0)	0 (0)	0 (0)	0 (0)
CTX-M	7 (1.1)	0 (0)	0 (0)	7 (0.8)	6 (0.9)	2 (2.7)	1 (1.2)	9 (1.1)
Atypical bacteria								
C. pneumoniae	1 (0.2)	0 (0)	0 (0)	1 (0.1)	0 (0)	0 (0)	0 (0)	0 (0)
L. pneumophila	2 (0.3)	0 (0)	0 (0)	2 (0.2)	0 (0)	0 (0)	0 (0)	0 (0)
M. pneumoniae	3 (0.5)	1 (0.6)	0 (0)	4 (0.5)	2 (0.3)	0 (0)	5 (6.2)	7 (0.8)
Viruses								
Adenovirus	7 (1.1)	1 (0.6)	0 (0)	8 (0.9)	12 (1.8)	1 (1.4)	3 (3.7)	16 (1.9)
Coronavirus	22 (3.3)	9 (5.7)	0 (0)	31 (3.7)	23 (3.4)	8 (11)	4 (4.9)	35 (4.2)
Human metapneumovirus	6 (0.9)	2 (1.3)	1 (4.8)	9 (1.1)	17 (2.5)	1 (1.4)	4 (4.9)	22 (2.6)
Human rhinovirus/enterovirus	47 (7.1)	17 (10.7)	0 (0)	64 (7.6)	69 (10.1)	18 (24.7)	25 (30.9)	112 (13.4)
Influenza A virus	13 (2.0)	2 (1.3)	0 (0)	15 (1.8)	9 (1.3)	3 (4.1)	4 (4.9)	16 (1.9)
Influenza B virus	6 (0.9)	0 (0)	1 (4.8)	7 (0.8)	11 (1.6)	1 (1.4	2 (2.5)	14 (1.7)
MERS-CoV^*a*^	0 (0)	0 (0)	0 (0)	0 (0)	0 (0)	0 (0)	0 (0)	0 (0)
Parainfluenza virus	17 (2.6)	1 (0.6)	0 (0)	18 (2.1)	25 (3.7)	0 (0)	5 (6.2)	30 (3.6)
Respiratory syncytial virus	4 (0.6)	0 (0)	0 (0)	4 (0.5)	38 (5.6)	2 (2.7)	8 (9.9)	48 (5.7)

a MERS-CoV is reported only in the BioFire PNplus panel product.

### Qualitative analysis of typical bacteria.

The performance characteristics of the PN panel for semiquantifiable bacterial targets compared to the reference method of qRefCx performed at the central lab are presented in Table 6. A specimen was considered positive for a particular organism by qRefCx when it was recovered and enumerated at a level greater than 3,162 (10^3.5^) CFU/ml approximated using dilution plating, which is equal to or greater than the PN panel reporting threshold of 10^3.5^ genomic copies/ml. The overall sensitivity for sputum samples ranged from 75% to 100%, and that for BAL specimens ranged from 85.7% to 100%. Sensitivity for A. calcoaceticus-A. baumannii, Moraxella catarrhalis, and Streptococcus agalactiae could not be calculated for BAL specimens due to limited detections by the qRefCx comparator method. Specificity for all analytes in both specimen types ranged from 88.9% to 99.5%.

**TABLE 6:** Qualitative BioFire PN panel performance for bacteria using qRefCx and SOC as comparators

Organism	Specimen^*a*^	qRefCx (10^3.5^ cutoff)				SOC^*b*^			
		Sensitivity		Specificity		Sensitivity		Specificity	
		TP/(TP+FN)	% (95% CI)	TN/(TN+FP)	% (95% CI)	TP/(TP+FN)	% (95% CI)	TN/(TN+FP)	% (95% CI)
A. calcoaceticus-A. baumannii complex	BAL	0/0		839/846	99.2 (98.3–99.6)	2/2	100 (34.2–100)	831/836	99.4 (98.6–99.7)
	SPU	10/11	90.9 (62.3–98.4)	807/825	97.8 (96.6–98.6)	12/15	80.0 (54.8–93.0)	789/805	98.0 (96.8–98.8)
E. cloacae complex	BAL	11/12	91.7 (64.6–98.5)	822/834	98.6 (97.5–99.2)	15/17	88.2 (65.7–96.7)	813/821	99.0 (98.1–99.5)
	SPU	11/12	91.7 (64.6–98.5)	803/824	97.5 (96.1–98.3)	12/13	92.3 (66.7–98.6)	788/807	97.6 (96.4–98.5)
E. coli	BAL	12/12	100 (75.8–100)	826/834	99.0 (98.1–99.5)	16/17	94.1 (73.0–99.0)	817/821	99.5 (98.8–99.8)
	SPU	23/24	95.8 (79.8–99.3)	787/812	96.9 (95.5–97.9)	20/22	90.9 (72.2–97.5)	771/798	96.6 (95.1–97.7)
H. influenzae	BAL	10/10	100 (72.2–100)	764/836	91.4 (89.3–93.1)	25/25	100 (86.7–100)	757/813	93.1 (91.2–94.7)
	SPU	16/18	88.9 (67.2–96.9)	727/818	88.9 (86.5–90.9)	30/32	93.8 (79.9–98.3)	715/788	90.7 (88.5–92.6)
K. aerogenes	BAL	6/7	85.7 (48.7–97.4)	832/839	99.2 (98.3–99.6)	8/11	72.7 (43.4–90.3)	823/827	99.5 (98.8–99.8)
	SPU	3/4	75.0 (30.1–95.4)	823/832	98.9 (98.0–99.4)	6/7	85.7 (48.7–97.4)	807/813	99.3 (98.4–99.7)
K. oxytoca	BAL	2/2	100 (34.2–100)	835/844	98.9 (98.0–99.4)	4/7	57.1 (25.0–84.2)	824/831	99.2 (98.3–99.6)
	SPU	9/9	100 (70.1–100)	817/827	98.8 (97.8–99.3)	7/8	87.5 (52.9–97.8)	801/812	98.6 (97.6–99.2)
K. pneumoniae group	BAL	15/15	100 (79.6–100)	819/831	98.6 (97.5–99.2)	18/19	94.7 (75.4–99.1)	810/819	98.9 (97.9–99.4)
	SPU	21/23	91.3 (73.2–97.6)	769/813	94.6 (92.8–95.9)	31/33	93.9 (80.4–98.3)	754/787	95.8 (94.2–97.0)
M. catarrhalis	BAL	0/0	–	817/846	96.6 (95.1–97.6)	9/9	100 (70.1–100)	810/829	97.7 (96.4–98.5)
	SPU	5/5	100 (56.6–100)	761/831	91.6 (89.5–93.3)	23/24	95.8 (79.8–99.3)	746/796	93.7 (91.8–95.2)
*Proteus* spp.	BAL	5/5	100 (56.6–100)	837/841	99.5 (98.8–99.8)	5/5	100 (56.6–100)	829/833	99.5 (98.8–99.8)
	SPU	15/15	100 (79.6–100)	813/821	99.0 (98.1–99.5)	6/7	85.7 (48.7–97.4)	797/813	98.0 (96.8–98.8)
P. aeruginosa	BAL	36/36	100 (90.4–100)	772/810	95.3 (93.6–96.6)	49/53	92.5 (82.1–97.0)	762/785	97.1 (95.6–98.0)
	SPU	103/106	97.2 (92.0–99.0)	673/730	92.2 (90.0–93.9)	115/122	94.3 (88.6–97.2)	654/698	93.7 (91.6–95.3)
S. marcescens	BAL	6/6	100 (61.0–100)	834/840	99.3 (98.5–99.7)	8/8	100 (67.6–100)	826/830	99.5 (98.8–99.8)
	SPU	26/27	96.3 (81.7–99.3)	782/809	96.7 (95.2–97.7)	25/29	86.2 (69.4–94.5)	763/791	96.5 (94.9–97.5)
S. aureus	BAL	46/47	97.9 (88.9–99.6)	729/799	91.2 (89.1–93.0)	69/75	92.0 (83.6–96.3)	717/763	94.0 (92.1–95.4)
	SPU	111/112	99.1 (95.1–99.8)	631/724	87.2 (84.5–89.4)	124/133	93.2 (87.6–96.4)	612/687	89.1 (86.5–91.2)
S. agalactiae	BAL	1/1	–	821/845	97.2 (95.8–98.1)	5/5	100 (56.6–100)	813/833	97.6 (96.3–98.4)
	SPU	9/9	100 (70.1–100)	793/827	95.9 (94.3–97.0)	10/10	100 (72.2–100)	778/810	96.0 (94.5–97.2)
S. pneumoniae	BAL	5/5	100 (56.6–100)	817/841	97.1 (95.8–98.1)	12/13	92.3 (66.7–98.6)	808/825	97.9 (96.7–98.7)
	SPU	16/16	100 (80.6–100)	785/820	95.7 (94.1–96.9)	10/13	76.9 (49.7–91.8)	768/807	95.2 (93.5–96.4)
S. pyogenes	BAL	2/2	100 (34.2–100)	838/844	99.3 (98.5–99.7)	5/5	100 (56.6–100)	831/833	99.8 (99.1–99.9)
	SPU	6/6	100 (61.0–100)	825/830	99.4 (98.6–997)	5/6	83.3 (43.6–97.0)	808/814	99.3 (98.4–99.7)

a SPU, sputum.

b Any result in subject medical record.

Compared to a quantitative reference culture, false-negative results were uncommon, with no more than 3 observed for any organism and 16 total among the 1,682 specimens tested. Comparatively, false-positive results were relatively common in both specimen types. The highest rates of false-positive detections were seen for the organisms most frequently detected: 163 total for both S. aureus and H. influenzae, 99 for M. catarrhalis, and 95 for P. aeruginosa.

Discrepancies between positive detection by PN panel and negative qRefCx culture report were evaluated by first determining if the organism was reported as negative because it was enumerated below the threshold of <10^3.5^ (3,162) CFU/ml set for culture. If discrepancies remained unresolved, the results of an independent molecular assay were considered. Finally, if discrepancies remained, the results from SOC testing at the individual sites were considered. Results of discrepancy analysis are shown in Table 7. A total of 875 discrepant false-positive results were observed between the PN panel and comparator qRefCx. A quarter (25.1%; 220/875) of the discrepancies between the PN panel and qRefCx were resolved as the organism being present but enumerated below the reference culture cutoff of 10^3.5^ CFU/ml. An additional 74.5% (652/875) were resolved using the results of an alternative molecular method or by evaluating the results of SOC culture. Among 16 specimens with false-negative results, evidence of the target organism was found in 10 specimens by molecular testing (9 specimens) or SOC culture (1 specimen); the false-negative results were attributed to low levels of organism in the specimen, i.e., at or below the PN panel reporting cutoff. Sequencing of bacterial isolates recovered from five remaining false-negative specimens indicated misidentification by the reference lab performing qRefCx (one A. baumannii isolate sequenced as Pseudomonas fluorescens, one H. influenzae isolate sequenced as Haemophilus haemolyticus, one K. aerogenes isolate sequenced as Hafnia paralvei, and two P. aeruginosa isolates sequenced as Pseudomonas denitrificans and Pseudomonas fluorescens). Investigation of the final false-negative K. pneumoniae result uncovered evidence of a specimen swap or paperwork error. Following discrepancy testing and analysis, only three false positives remained unresolved. No evidence of nonspecific amplification was observed for the PN panel.

**TABLE 7:** BioFire PN panel discrepancy investigation for detection of bacteria

Analyte	Specimen^*a*^	No. of results													
		False positive vs. qRefCx (investigative method)					False negative vs. qRefCx (investigative method)				False negative vs. SOC culture (SOC quantity^*b*^)				
		Total	BQ^*c*^	Molec^*d*^	SOC	rTP^*e*^	Tot	Molec^*d*^	SOC	cFN^*f*^	Total	Few	Mod	Many	UQ
A. calcoaceticus-A. baumannii complex	BAL	7	1	6		7	0				0				
	SPU	18		17	1	18	1			0^*g*^	3	0	2	1	0
E. cloacae complex	BAL	12	6	5		11	1	1		1	2	0	2	0	0
	SPU	21	4	17		21	1	1		1	1	1	0	0	0
E. coli	BAL	8	6	2		8	0				1	1	0	0	0
	SPU	25	6	19		25	1	1		1	2	1	0	0	1
H. influenzae	BAL	72	7	64	1	72	0				0				
	SPU	91	4	85	2	91	2	1		1^*g*^	2	0	1	1	0
K. aerogenes	BAL	7	4	3	1	8	1		1	1	3	2	0	1	0
	SPU	9	3	6		9	1			0^*g*^	1	0	0	0	1
K. oxytoca	BAL	9	3	6		9	0				3	2	0	1	0
	SPU	10	3	7		10	0				1	0	0	0	1
K. pneumoniae group	BAL	12	7	5		12	0				1	1	0	0	0
	SPU	44	15	28		43	2	1		1^*h*^	2	1	0	0	1
M. catarrhalis	BAL	29	2	27		29	0				0				
	SPU	70	1	68	1	70	0				1	1	0	0	0
*Proteus* spp.	BAL	4	3	1		4	0				0				
	SPU	8	2	6		8	0				1	0	0	0	1
P. aeruginosa	BAL	38	19	19		38	0				4	3	0	0	1
	SPU	57	21	35	1	57	3	1		1^*g*^	7	5	0	1	1
S. marcescens	BAL	6	4	2		6	0				0				
	SPU	27	7	19		26	1	1		1	4	2	0	0	2
S. aureus	BAL	70	29	38	2	69	1	1		1	6	5	1	0	0
	SPU	93	43	46	4	93	1	1		1	9	7	0	0	2
S. agalactiae	BAL	24	7	17		24	0				0				
	SPU	34	5	29		34	0				0				
S. pneumoniae	BAL	24	5	19		24	0				1	1	0	0	0
	SPU	35	1	34		35	0				3	1	0	1	1
S. pyogenes	BAL	6	2	4		6	0				0				
	SPU	5	0	5		5	0				1	0	1	0	0
Total		875	220	639	13	872	16	9	1	10	59	34	7	6	12

a SPU, sputum.

b Reported organism quantity in subject medical record. “Few” corresponds to values of <10,000 CFU/ml and the descriptions “few,” “1+,” “light growth,” “rare,” and “1 colony”; “Mod” corresponds to values of 10,000 to <100,000 CFU/ml and the descriptions “moderate,” “2+,” and “3+”; “Many” corresponds to values of ≥100,000 and the descriptions “many,” “heavy growth,” and “4+.” UQ, unable to quantify (quantity was given in relative terms, e.g., “listed first” or “least of three”).

c BQ, organism present in qRefCx but enumerated below the quantification threshold of 10^3.5^ (3,125) CFU/ml.

d Molec, molecular.

e rTP, resolved true positive; evidence of organism presence confirming BioFire PN panel correct result.

f cFN, confirmed false negative; evidence of organism presence confirming BioFire PN panel incorrect result.

g Isolates misidentified by the central lab (see the text).

h Evidence of specimen swap at central lab (see the text).

An additional qualitative analysis of the PN panel semiquantitative results for bacteria was performed by comparing them to SOC culture results (Table 6). In this analysis, an organism was considered positive by SOC if a result for the particular bacterial analyte was entered in the subject’s medical record, regardless of any quantity information that may have been indicated. While specificity by this method is similar to that of the qRefCx culture method, sensitivity is lower for some analytes. This was attributed to the fact that the analysis considered an analyte positive by SOC if it was reported at any level. While some organisms were reported in subject medical records with a numerical quantity, most were reported with qualitative descriptions such as “few,” “most abundant,” “2+,” etc., and therefore, this information could not uniformly be converted to a numeric value equivalent to the PN panel reporting threshold. An investigation of 59 PN panel false-negative results relative to SOC revealed that the majority (34; 57.6%) had been reported as being present at a low level (i.e., “few”) and may have been present below the PN panel cutoff. Thirteen (22%) were reported at higher levels, and 12 (20.3%) could not be categorized because the reported quantities were described in relative terms (e.g., “listed first” or “least of three”).

### Quantitative analysis of typical bacteria.

PN panel semiquantitative results were compared to qRefCx results (Table 8) using the following analysis. qRefCx results for each organism were stratified into 1-log_10_ ranges (e.g., 10^4^ to <10^5^, 10^5^ to <10^6^, etc.) (Table 8). The PN panel bin result for a particular analyte was considered concordant if the reported bin value was at either end of that range (e.g., a qRefCx value of 35,000 CFU/ml, or 3.5 × 10^4^, which falls between 10^4^ and 10^5^, was concordant with a PN panel bin result of either 10^4^ or 10^5^). Concordance was low for qRefCx values below 10^6^ CFU/ml, with overall values ranging from 3.1% to 38.9% for both specimen types (Table 8, “=” columns). However, when qRefCx values were above 10^6^, PN panel concordance was 90.9% to 100% for both specimen types. When discrepant results were examined for a particular concentration range, there were very few instances where the PN panel result was “not detected” or was a value lower than that from qRefCx (Table 8, “ND” and “<” columns). However, the PN panel reported organism levels higher than the qRefCx range for 58.9 to 93.8% of specimens with qRefCx values below 10^6^ (Table 8, “>” columns). Performance was similar for all organisms.

**TABLE 8:** BioFire PN panel semiquantitative concordance with qRefCx

Target	Specimen	No. of samples with result/total (%) for qRefCx range (CFU/ml) [BioFire PN panel equivalent bin]^*a*^												
		10^3.5^ to <10^4^ [10^4^]			10^4^ to <10^5^ [10^4^ or 10^5^]			10^5^ to <10^6^ [10^5^ or 10^6^]			10^6^ to <10^7^ [10^6^ or ≥10^7^]		≥10^7^ [≥10^7^]	
		ND	=	>	<	=	>	<	=	>	<	=	<	=
A. calcoaceticus-A. baumannii complex	BAL		0/0 ()			0/0 ()			0/0 ()			0/0 ()		0/0 ()
	SPU		1/5 (20.0)	4		1/1 (100)		1	0/2 (0)	1		3/3 (100)		0/0 ()
E. cloacae complex	BAL		0/1 (0)	1	1	1/7 (14.3)	5		0/3 (0)	3		0/0 ()		1/1 (100)
	SPU		0/1 (0)	1	1	3/6 (50.0)	2		1/3 (33.3)	2		1/1 (100)		1/1 (100)
E. coli	BAL		0/4 (0)	4		1/7 (14.3)	6		0/0 ()			0/0 ()		1/1 (100)
	SPU		2/3 (66.7)	1	1	1/13 (7.7)	11		2/5 (40.0)	3		1/1 (100)		2/2 (100)
H. influenzae	BAL		0/2 (0)	2		0/2 (0)	2		0/5 (0)	5		0/0 ()		1/1 (100)
	SPU		0/0 ()		1	0/12 (0)	11	1	1/4 (25.0)	2		2/2 (100)		0/0 ()
K. aerogenes	BAL		0/1 (0)	1	1	1/2 (50.0)			0/1 (0)	1	1	2/3 (66.7)		0/0 ()
	SPU		1/1 (100)		1	0/1 (0)			0/1 (0)	1		1/1 (100)		0/0 ()
K. oxytoca	BAL		0/0 ()			1/2 (50.0)	1		0/0 ()			0/0 ()		0/0 ()
	SPU		1/3 (33.3)	2		0/3 (0)	3		2/2 (100)			1/1 (100)		0/0 ()
K. pneumoniae group	BAL		0/5 (0)	5		3/5 (60.0)	2		0/0 ()			2/2 (100)		3/3 (100)
	SPU	1	3/6 (50.0)	2		2/7 (28.6)	5		5/7 (71.4)	2		2/2 (100)	1	0/1 (0)
M. catarrhalis	BAL		0/0 ()			0/0 ()			0/0 ()			0/0 ()		0/0 ()
	SPU		0/1 (0)	1		0/1 (0)	1		0/1 (0)	1		1/1 (100)		1/1 (100)
*Proteus* spp.	BAL		0/1 (0)	1		1/1 (100)			0/1 (0)	1		1/1 (100)		1/1 (100)
	SPU		0/1 (0)	1		5/10 (50.0)	5		2/3 (66.7)	1		1/1 (100)		0/0 ()
P. aeruginosa	BAL		0/4 (0)	4		2/15 (13.3)	13		1/13 (7.7)	12		2/2 (100)		2/2 (100)
	SPU	2	3/15 (20.0)	10	1	7/42 (16.7)	34		5/24 (20.8)	19		19/19 (100)		6/6 (100)
S. marcescens	BAL		0/1 (0)	1		1/4 (25.0)	3		0/1 (0)	1		0/0 ()		0/0 ()
	SPU		0/4 (0)	4	1	4/12 (33.3)	7		3/6 (50.0)	3		4/4 (100)		1/1 (100)
S. aureus	BAL	1	0/11 (0)	10		0/19 (0)	19		1/9 (11.1)	8		5/5 (100)		3/3 (100)
	SPU	1	1/18 (5.6)	16		11/45 (24.4)	34		13/28 (46.4)	15	1	12/13 (92.3)	1	7/8 (87.5)
S. agalactiae	BAL		0/0 ()			0/1 (0)	1		0/0 ()			0/0 ()		0/0 ()
	SPU		0/3 (0)	3		1/5 (20.0)	4		0/1 (0)	1		0/0 ()		0/0 ()
S. pneumoniae	BAL		1/2 (50.0)	1		1/2 (50.0)	1		0/1 (0)	1		0/0 ()		0/0 ()
	SPU		2/3 (66.7)	1		2/6 (33.3)	4		0/2 (0)	2		4/4 (100)		1/1 (100)
S. pyogenes	BAL		0/0 ()			2/2 (100)			0/0 ()			0/0 ()		0/0 ()
	SPU		0/0 ()			0/3 (0)	3		1/1 (100)			1/1 (100)		1/1 (100)
Overall	BAL	1/32 (3.1)	1/32 (3.1)	30/32 (93.8)	2/69 (2.9)	14/69 (20.3)	53/69 (76.8)	0/34 (0)	2/32 (5.9)	32/34 (94.1)	1/13 (7.7)	12/13 (92.3)	0/12 (0)	12/12 (100)
	SPU	4/64 (6.2)	14/64 (21.9)	46/64 (71.9)	6/167 (3.6)	37/167 (22.2)	124/167 (74.3)	2/90 (2.2)	35/90 (38.9)	53/90 (58.9)	1/54 (1.9)	53/54 (98.1)	2/22 (6.1)	20/22 (90.9)

a Concordance is indicated as follows: ND, not detected by BioFire PN panel; <, detected by the PN panel but reported in a lower bin not concordant with qRefCx (or not detected); =, detected by the PN panel in a bin concordant with qRefCx; >, detected by the PN panel but reported in a higher bin not concordant with qRefCx.

### S. aureus *mecA*/*mecC* and MREJ determinants.

When S. aureus is detected on the PN panel, it is accompanied by a result for the detection of *mecA* and *mecC*. These genes encode a penicillin-binding protein (PBP2a) that has low affinity for beta-lactams and are carried on a chromosomally integrated mobile genetic element called the staphylococcal cassette chromosome *mec* (SCC*mec*), which may be found in many *Staphylococcus* spp. To distinguish between methicillin-resistant S. aureus (MRSA) or codetection of methicillin-sensitive S. aureus (MSSA) and another *Staphylococcus* sp. carrying the SCC*mec* cassette and *mecA*/*mecC*, the PN panel contains an additional assay that amplifies the SCC*mec* right-extremity junction (MREJ), which links the SCC*mec* cassette to the S. aureus genome and indicates MRSA. S. aureus was detected in 116 BAL and 204 sputum samples. The PN panel *mecA*/*mecC* and MREJ “detected” results for these specimens were compared to results of molecular testing performed directly from the specimen, with PPA and NPA of 88.9% and 91.4% for BAL and 95.9% and 87.5% for sputum, respectively (Table 9). Investigation of the 28 false-positive and false-negative specimens using independent molecular methods found evidence of *mecA*/*mecC* and MREJ in 27 of them (Table 10; one specimen could not be investigated due to lack of remaining volume). A review of AST testing performed on S. aureus isolates recovered by SOC and qRefCx methods (data not shown) revealed that many of the specimens with discrepant results were polymicrobial with both MRSA and MSSA. Some specimens were polymicrobial with other methicillin-resistant *Staphylococcus* spp. (i.e., organisms carrying *mecA*/*mecC*) and an MSSA isolate which may have carried an empty SCC*mec* cassette (and thus was positive for MREJ and *mecA*/*mecC* but was not MRSA). When these different organisms are present together at near-LoD levels in polymicrobial specimens, differential detection by the PN panel and reference methods (including phenotypic AST) leads to discordant results (14). The MREJ sequence from one false-negative specimen was found to contain a sequence that is nonreactive to the PN panel MREJ primers; this limitation is noted in the product instructions for use (11).

**TABLE 9:** Performance of the BioFire PN panel for AMR determinants compared to independent PCR/sequencing

Analyte^*a*^	Specimen	Positive percent agreement		Negative percent agreement	
		TP/(TP+FN)	% (95% CI)	TN/(TN+FP)	% (95% IC)
*mecA*/*mecC* + MREJ	BAL	40/45	88.9 (76.5–95.2)	64/70	91.4 (82.5–96.0)
	SPU	94/98	95.9 (90.0–98.4)	91/104	87.5 (79.8–92.5)
KPC	BAL	2/2	100 (34.2–100)	148/149	99.3 (96.3–99.9)
	SPU	7/7	100 (64.6–100)	284/284	100 (98.7–100)
NDM	BAL	0/1	0	149/150	99.3 (96.3–99.9)
	SPU	0/0		291/291	100 (98.7–100)
OXA-48	BAL	0/0		151/151	100 (97.5–100)
	SPU	0/0		291/291	100 (98.7–100)
VIM	BAL	0/0		151/151	100 (97.5–100)
	SPU	1/1	100 (20.7–100)	289/290	99.7 (98.1–99.9)
IMP	BAL	0/0		151/151	100 (97.5–100)
	SPU	0/0		291/291	100 (98.7–100)
CTX-M	BAL	6/7	85.7 (48.7–97.4)	144/144	100 (97.4–100)
	SPU	8/10	80 (49.0–94.3)	280/281	99.6 (98.0–99.9)

a Reported only when an applicable host organism is also detected by the BioFire PN panel (see Table 1, footnotes b, c, and d).

**TABLE 10:** BioFire PN panel AMR discrepancy investigation

Analyte	Specimen^*a*^	No. of samples with result			
		False positive		False negative	
		Total	rTP^*b*^	Total	cFN^*c*^
AMR markers					
*mecA*/*mecC* + MREJ	BAL	6	5^*d*^	5	5
	SPU	13	13	4	4
KPC	BAL	1	1		
	SPU				
NDM	BAL	1	0	1	1
	SPU				
VIM	BAL				
	SPU	1	0		
CTX-M	BAL			1	1
	SPU	1	0	2	1
Viruses					
Adenovirus	BAL				
	SPU	2	1	4	4
Coronavirus	BAL	13	8	3	2
	SPU	6	3	4	4
Human metapneumovirus	BAL	1	0		
	SPU	1	0	1	1
Rhinovirus/enterovirus	BAL	11	8	2	2
	SPU	13	12		
Influenza A virus	BAL	3	3		
	SPU	3	2		
Influenza B virus	BAL	1	1	1	1
	SPU	2	0		
Parainfluenza virus	BAL	2	2	2	2
	SPU	2	1	1	1
RSV	BAL				
	SPU	4	4		
Atypical bacteria					
C. pneumoniae	BAL	1	0		
	SPU				
L. pneumophila	BAL				
	SPU			1	1
M. pneumoniae	BAL	1	0		
	SPU			1	0
Total		89	64	33	30

a SPU, sputum.

b rTP, resolved true positive; evidence of AMR presence by independent molecular method confirming BioFire PN panel correct result.

c cFN, confirmed false negative; evidence of AMR presence by independent molecular method confirming BioFire PN panel incorrect result.

d Insufficient leftover volume for discrepancy investigation of one specimen.

### Carbapenemase and extended-spectrum beta-lactamase AMR performance.

The PN panel includes assays for six AMR genes associated with carbapenem and extended-spectrum beta-lactam resistance that are reported for select Gram-negative bacteria. These genes are reported as “N/A” if no applicable host organism is detected in the specimen (Table 1, footnotes b, c, and d). CTX-M and KPC were the most commonly detected AMR targets in both BAL and sputum samples (Table 5). VIM was detected in two sputum samples, NDM was detected in one BAL specimen, and IMP and OXA-48-like genes were not detected. The comparator method for AMR gene performance was an independent molecular method performed on the specimen (a comparison of PN panel AMR gene detection to phenotypic AST of recovered isolates may be found in the PN panel instructions for use [11]; however, this method was not used as a primary comparator because the PN panel detected more organisms than were recovered by culture [Table 7] and also because phenotypic antimicrobial susceptibility may be conferred by mechanisms other than the genes reported by the PN panel, thus confounding interpretation of such results). KPC detection had a performance of 100% PPA in both sample types, with 100% NPA in sputum and 99.3% NPA in BAL. CTX-M detection had a PPA of 85.7% in BALs and 80% in sputum samples and NPA of 100% in BALs and 99.6% in sputum samples. Of two VIM detections in sputum specimens, one was true positive (100% PPA) and one false positive (resulting in 99.7% NPA). There was one NDM detection in BAL, but it was a false positive (99.3% NPA); the comparator method also detected a single NDM, but this was not observed by the PN panel and was considered to be a false negative (0% PPA). Discrepancy investigation with independent molecular methods found evidence of the AMR gene in several of the discrepant positive and negative results (resolved true positives and confirmed false negatives) (Table 10), suggesting analyte presence near the LoD of both the PN panel and comparator assays. As overall prevalence of these resistance gene markers was low in the study population, contrived specimens were utilized to further demonstrate the positive and negative percent agreement of the resistance targets as described in the product instructions for use (11).

### Analysis of viruses and atypical bacteria.

The overall performance of atypical bacterial and viral targets on the PN panel is summarized in Table 11. The PPA, NPA, and 95% CI were calculated compared to comparator methods of PCR and sequencing. PPA for MERS-CoV could not be calculated, as no detections occurred during the course of this study; NPA was 100%. The PPA for 2/8 targets was 100% for both BAL and sputum. The lowest PPA for sputum was 76.5% for adenovirus, with discordant results observed among all age groups; PPA for adenovirus was 100% in BAL. For BAL specimens, the lowest observed PPA was 85.7% for coronavirus. Atypical bacterial detections were rare overall, with the most frequent coming from Mycoplasma pneumoniae, which demonstrated PPA and NPA of 87.5% to 100%. Discordant results were attributed in most cases to low levels of analyte, i.e., at or near the LoD; investigation with independent molecular assays found evidence of analyte presence in the majority of false-positive and nearly all false-negative specimens (Table 10).

**TABLE 11:** Performance of the BioFire PN panel for atypical bacteria and viruses compared to independent PCR and sequencing

Analyte	Source^*a*^	Positive percent agreement		Negative percent agreement	
		TP/(TP+FN)	% (95% CI)	TN/(TN+FP)	95% CI
Atypical bacteria					
C. pneumoniae	BAL	0/0		844/845	99.9 (99.3–100)
	SPU	0/0		835/835	100 (99.5–100)
L. pneumophila	BAL	2/2	100 (34.2–100)	833/833	100 (99.5–100)
	SPU	0/1		826/826	100 (99.5–100)
M. pneumoniae	BAL	3/3	100 (43.9–100)	841/842	99.9 (99.3–100)
	SPU	7/8	87.5 (52.9–97.8)	827/827	100 (99.5–100)
Viruses					
Adenovirus	BAL	8/8	100 (67.6–100)	837/837	100 (99.5–100)
	SPU	13/17	76.5 (52.7–90.4)	815/817	99.8 (99.1–99.9)
Coronavirus	BAL	18/21	85.7 (65.4–95.0)	810/823	98.4 (97.3–99.1)
	SPU	28/32	87.5 (71.9–95.0)	796/802	99.3 (98.4–99.7)
Human metapneumovirus	BAL	8/8	100 (67.6–100)	836/837	99.9 (99.3–100)
	SPU	20/21	95.2 (77.3–99.2)	812/813	99.9 (99.3–100)
Rhinovirus/enterovirus	BAL	52/54	96.3 (87.5–99.0)	771/782	98.6 (97.5–99.2)
	SPU	96/96	100 (96.2–100)	717/730	98.2 (97.0–99.0)
Influenza A virus	BAL	10/10	100 (72.2–100)	830/833	99.6 (98.9–99.9)
	SPU	13/13	100 (77.2–100)	819/822	99.6 (98.9–99.9)
Influenza B virus	BAL	5/6	83.3 (43.6–97)	837/838	99.9 (99.3–100)
	SPU	12/12	100 (75.8–100)	821/823	99.8 (99.1–99.9)
MERS-CoV	BAL	0/0		846/846	100 (99.5–100)
	SPU	0/0		836/836	100 (99.5–100)
Parainfluenza virus	BAL	16/18	88.9 (67.2–96.9)	824/826	99.8 (99.1–99.9)
	SPU	28/29	96.6 (82.8–99.4)	804/806	99.8 (99.1–99.9)
RSV	BAL	3/3	100 (43.9–100)	841/841	100 (99.5–100)
	SPU	43/43	100 (91.8–100)	787/791	99.5 (98.7–99.8)

a SPU, sputum.

## DISCUSSION

Lower respiratory tract infections can be caused by a wide range of pathogens. Commonly, multiple diagnostic tests, including culture, molecular detection, and antigen detection, may be ordered to aid in the diagnosis of these infections. While awaiting the results of diagnostic testing, many patients are placed on broad-spectrum antibiotic therapy. In the absence of a clear diagnosis, antibiotic de-escalation may be delayed or rarely initiated. Furthermore, it is estimated that 30% of cases of community-acquired pneumonia (CAP) have no identified etiological cause (15). Due to the insensitivity of culture, the Infectious Disease Society of America (IDSA) does not recommend culture of lower respiratory tract specimens for ambulatory patients with CAP, owing to the low yield of culture and resulting minimal impact on patient care (16). Culture remains the recommendation for patients with severe CAP and for hospitalized patients with pneumonia. Molecular methods for a variety of infectious processes have shown a clear increase in sensitivity and rapid turnaround times (17–19).

This evaluation of the PN panel demonstrates the performance of this multiplex IVD test in selected analytical validation studies and a large prospective set of residual samples collected from a geographically and demographically diverse patient population. With the exception of a few targets that were not circulating in the population during the study period (e.g., MERS-CoV, Chlamydia pneumoniae, and some AMR genes), considerable numbers of most analytes were detected in both specimen types, allowing the determination of sensitivity/PPA and specificity/NPA. The panel detects 15 routinely encountered Gram-positive and Gram-negative pathogens. The sensitivity of this assay was >95% for 10 of these analytes in both BAL and sputum specimens. Sensitivities for the other five organisms ranged from 75% to 91.7%. Specificity for all targets in both specimen types was >91%.

The most challenging observation from these data is the discrepancy between the PN panel and culture for the detection and quantification of bacterial analytes. As shown in Table 6, the PN panel demonstrated a lower specificity for bacterial analytes that were commonly detected (S. aureus and P. aeruginosa) than qRefCx. This finding correlates with those for other diagnostic assays that have been developed for the detection of lower respiratory tract pathogens (20), highlighting the increased sensitivity of molecular methods compared to culture for common pathogens. This is attributed to multiple factors. While culture remains the gold standard in the diagnosis of bacterial respiratory tract infections, it may be difficult to accurately recover all pathogens in clinical samples, as the organisms are in a complex matrix. In addition, culture results would be more affected by host immune response and prior antibiotic usage. Culture is also subject to the criteria of each laboratory and to interpretation by the technologists examining those cultures. The panel is more robust against variability than could be attributed to the sample matrix, different techniques among laboratories, and recovery of more fastidious organisms. A potential drawback of molecular methods is the detection of nonviable organisms, but that may aid in the de-escalation of antibiotics in the absence of organism detection by culture in patients with prior antibiotic exposure.

The PN panel was shown to reliably detect and quantify bacterial genomes (Table 1) and was also shown to be able to detect the relative abundance of each target in contrived polymicrobial specimens (Table 2). Further work is needed to determine if detection of organisms at low abundances in the PN panel that are not identified in culture is significant for patient outcomes. Preliminary work done concurrently during this trial demonstrated the potential use of this panel as a diagnostic tool (12).

A challenge of interpretation of respiratory cultures or results from molecular diagnostics like the PN panel is determining if the organisms detected are clinically significant. Many clinically significant organisms may be normal flora of the oropharyngeal tract, particularly when they are present in a lower abundance. In the culture of lower respiratory tract specimens, it is important to report significant amounts of pathogens from sputum (often defined as presence of the organism in the second or third quadrant) or ≥10^4^ CFU in BAL specimens. Previous studies have shown that quantitative PCR can be a means to differentiate commensalism from pathogenicity by looking at the nucleic acid burden (21). To promote adherence to current IDSA recommendations, the PN panel reports only organisms that are detected at >10^3.5^ copies/ml. It then places the positive results into semiquantitative bins of 10^4^, 10^5^, 10^6^, and ≥10^7^. In culture, it may be difficult to find significant organisms present in lower, but still clinically relevant, amounts in the presence of large numbers of other pathogenic or commensal organisms. The PN panel demonstrated that detection of organisms near the limit of detection was not influenced by the presence of a high burden of other organisms (Table 2).

The data collected in this prospective study demonstrate that the PN panel is sensitive for the detection of bacterial analytes, as only a limited number of false negatives were observed when the PN panel was compared to qRefCx or SOC (Table 6). This indicates that the panel cutoff of 10^3.5^ genomes/ml is appropriate. The false negatives were attributed to organisms present in numbers below the lowest PN panel bin due to misidentifications at the central reference lab.

The PN panel is additionally able to provide preliminary indication of potential antimicrobial susceptibility data for some commonly encountered pathogens via detection of selected AMR genes. Detection of *mecA*/*mecC* in conjunction with MREJ was shown to have high PPA and NPA with an independent molecular method, ranging from 87.5% to 95.9%. The panel is also able to detect CTX-M-type extended-spectrum beta-lactamases (ESBLs). Since the emergence of CTX-M-type ESBLs in the 1990s, these enzymes have become the most prevalent type of ESBL in a variety of settings throughout the world (22–24). CTX-M-type ESBLs are most prominent in E. coli and *Klebsiella* spp.; E. coli strains carrying CTX-M are prominent causes of community-onset urinary tract and bloodstream infections. CTX-M results are reported when any member of the family *Enterobacteriaceae*, Acinetobacter spp., or P. aeruginosa is detected, as these organisms have all been reported to potentially harbor ESBLs.

The PN panel may provide actionable information on antimicrobial susceptibility for some key organisms. However, appropriate antimicrobial therapy for many targets, particularly in areas where resistance is common, may require follow-up culture and susceptibility testing. This is especially true for organisms with mutation-based resistance, such as S. pneumoniae and P. aeruginosa. Implementation of these panels for routine clinical testing still requires additional culture or appropriate follow-up by the performing laboratories to ensure thorough evaluation of AST phenotypes.

Routine detection of viral analytes and atypical bacteria in upper respiratory tract specimens has been demonstrated on previous BioFire respiratory panels. This panel demonstrates performance attributes similar to those of the existing panels (25, 26). A notable difference with this panel is the combined identification of viral subtypes that are reported distinctly in other molecular diagnostic tests (e.g., “coronavirus” as a whole, rather than specific identification of HKU1, OC43, etc., or “influenza A virus” with no additional subtype information). While some of these data may be useful for epidemiological purposes, they should not influence treatment and patient care. The PN panel should have similar if not expanded clinical utility in these populations, facilitating faster access to appropriate treatment and improved clinical outcomes (27, 28). The panel also exceeds the utility of previous respiratory panels with the inclusion of Legionella pneumophila and the ability to detect a variety of serogroups (11). The PN panel provides a method with improved sensitivity for the diagnosis of Legionnaires’ disease, which is estimated to account for 2% to 6% of CAP. The current standard is a urine antigen test, which has a sensitivity of only 80% and is limited to detection of serogroup 1, while studies have shown that the use of PCR has improved sensitivity over the current gold standard (29).

The results for MERS-CoV are masked in the PN panel product that is FDA cleared and available in the United States. This analyte is reported in the BioFire PNplus panel, which is sold outside the United States and has also been cleared by the FDA with a modified intended use to specifically aid in the differential diagnosis of MERS-CoV infections only in cases meeting MERS-CoV clinical and/or epidemiological criteria.

The PN panel is intended for the use of both sputum and BAL fluid. Concurrent bacterial cultures have shown high rates of correlation between sputum and BAL specimens (30). While viral detection is traditionally done with nasopharyngeal samples, studies comparing use of nasopharyngeal swabs and BAL specimens using the BioFire FilmArray Respiratory (RP) panel (an off-label use of the product) have displayed high levels of correlation, with BAL specimens generally having a higher diagnostic yield (31, 32).

A weakness of this study was that a majority of the specimens enrolled were from hospitalized patients, but this likely reflects the severity of illness in this population and adherence to guidelines suggesting that diagnostic testing is not warranted in ambulatory patients. The data from this study indicate that specimens collected from hospitalized patients and those in outpatient settings had similar incidences of most analytes.

The use of a panel that provides sensitive and specific detection of respiratory tract pathogens has been shown to improve patient outcomes and is a recommended tool for antimicrobial stewardship initiatives (33–35). The PN panel expands on these existing technologies to provide an easy-to-use, rapid sample-to-answer platform that can detect viral entities and atypical bacteria known to cause pneumonia, in addition to providing a semiquantitative result for 15 commonly encountered bacterial analytes. An earlier study using an RUO version of the PN panel on BAL from patients suspected of having ventilator-associated pneumonia concluded that the panel would provide data that could guide appropriate management in this patient population (36).

The occurrence and impact of viral and bacterial coinfections in pneumonia are not well characterized, but recent studies have shown that coinfection is not unusual in community-acquired pneumonia in adults and was responsible for higher morbidity and mortality (37). Therefore, it is anticipated that the PN panel could significantly affect the management of patients with coinfections.

Current algorithms for the diagnosis of pneumonia can include multiple methods; molecular methods are most common for viral agents and many atypical bacteria, and culture remains the gold standard for the diagnosis of bacterial pneumonia. Culture suffers from lower sensitivity than molecular methods, in addition to variable methods of interpretation and reporting among and within an institution. Culture can also take an average of 48 to 72 h for actionable results to become available. Implementation of the PN panel will require consideration of appropriate test utilization in individual patient populations, but it has the potential to be a powerful decision-making tool for patient management. This panel could be utilized for rapid de-escalation or initiation of antibiotics and promoting improved patient care outcomes. Further studies are needed to evaluate the clinical impact of this panel and the significance of molecular detection in the absence of culture confirmation. Some of the data from this trial have been examined to determine the potential impact on patient care (12).
